# Supplementary material for: Heterogeneous multimeric structure of isocitrate lyase in complex with succinate and itaconate provides novel insights into its inhibitory mechanism
Source: PLoS One. 2021 May 5;16(5):e0251067. doi: 10.1371/journal.pone.0251067 (PMC8099091; doi:10.1371/journal.pone.0251067)
Supplement: S1 File — (PDF) [file pone.0251067.s001.pdf]

Full wwPDB X-ray Structure Validation Report (i)

Aug 7, 2020 – 09:08 AM JST

PDB ID	:	7CP1
Title	:	Crystal structure of isocitrate lyase in complex with succinate and itaconate
Deposited on	:	2020-08-05
Resolution	:	2.58 Å(reported)

This is a Full wwPDB X-ray Structure Validation Report.

This report is produced by the wwPDB biocuration pipeline after annotation of the structure.

We welcome your comments at *validation@mail.wwpdb.org*
A user guide is available at
https://www.wwpdb.org/validation/2017/XrayValidationReportHelp
with specific help available everywhere you see the (i) symbol.

CONFIDENTIAL VALIDATION REPORT

The following versions of software and data (see references (i)) were used in the production of this report:

MolProbity	:	4.02b-467
Mogul	:	1.8.5 (274361), CSD as541be (2020)
Xtriage (Phenix)	:	1.13
EDS	:	2.13.1
buster-report	:	1.1.7 (2018)
Percentile statistics	:	20191225.v01 (using entries in the PDB archive December 25th 2019)
Refmac	:	5.8.0158
CCP4	:	7.0.044 (Gargrove)
Ideal geometry (proteins)	:	Engh & Huber (2001)
Ideal geometry (DNA, RNA)	:	Parkinson et al. (1996)
Validation Pipeline (wwPDB-VP)	:	2.13.1

1 Overall quality at a glance (i)

The following experimental techniques were used to determine the structure:
X-RAY DIFFRACTION

The reported resolution of this entry is 2.58 Å.

Percentile scores (ranging between 0-100) for global validation metrics of the entry are shown in the following graphic. The table shows the number of entries on which the scores are based.

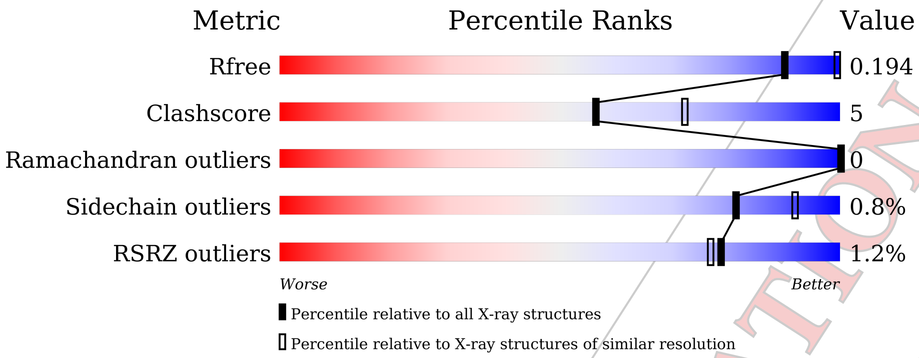

Metric	Whole archive (#Entries)	Similar resolution (#Entries, resolution range(Å))
R_{free}	130704	3676 (2.60-2.56)
Clashscore	141614	4049 (2.60-2.56)
Ramachandran outliers	138981	3979 (2.60-2.56)
Sidechain outliers	138945	3979 (2.60-2.56)
RSRZ outliers	127900	3614 (2.60-2.56)

The table below summarises the geometric issues observed across the polymeric chains and their fit to the electron density. The red, orange, yellow and green segments on the lower bar indicate the fraction of residues that contain outliers for >=3, 2, 1 and 0 types of geometric quality criteria respectively. A grey segment represents the fraction of residues that are not modelled. The numeric value for each fraction is indicated below the corresponding segment, with a dot representing fractions <=5% The upper red bar (where present) indicates the fraction of residues that have poor fit to the electron density. The numeric value is given above the bar.

Mol	Chain	Length	Quality of chain
1	A	436	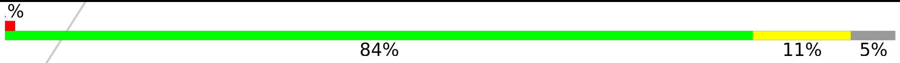
1	B	436	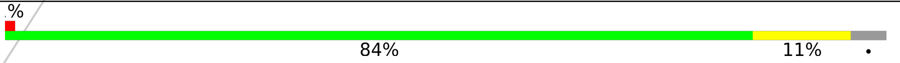

CONFIDENTIAL VALIDATION REPORT

2 Entry composition (i)

There are 5 unique types of molecules in this entry. The entry contains 7102 atoms, of which 0 are hydrogens and 0 are deuteriums.

In the tables below, the ZeroOcc column contains the number of atoms modelled with zero occupancy, the AltConf column contains the number of residues with at least one atom in alternate conformation and the Trace column contains the number of residues modelled with at most 2 atoms.

- Molecule 1 is a protein called Isocitrate lyase.

Mol	Chain	Residues	Atoms	ZeroOcc	AltConf	Trace
1	A	415	Total C N O S<br>3211 2020 559 623 9	0	0	0
1	B	417	Total C N O S<br>3231 2033 561 628 9	0	0	0

There are 16 discrepancies between the modelled and reference sequences:

Chain	Residue	Modelled	Actual	Comment	Reference
A	429	LEU	-	expression tag	UNP P9WKK7
A	430	GLU	-	expression tag	UNP P9WKK7
A	431	HIS	-	expression tag	UNP P9WKK7
A	432	HIS	-	expression tag	UNP P9WKK7
A	433	HIS	-	expression tag	UNP P9WKK7
A	434	HIS	-	expression tag	UNP P9WKK7
A	435	HIS	-	expression tag	UNP P9WKK7
A	436	HIS	-	expression tag	UNP P9WKK7
B	429	LEU	-	expression tag	UNP P9WKK7
B	430	GLU	-	expression tag	UNP P9WKK7
B	431	HIS	-	expression tag	UNP P9WKK7
B	432	HIS	-	expression tag	UNP P9WKK7
B	433	HIS	-	expression tag	UNP P9WKK7
B	434	HIS	-	expression tag	UNP P9WKK7
B	435	HIS	-	expression tag	UNP P9WKK7
B	436	HIS	-	expression tag	UNP P9WKK7

- Molecule 2 is SUCCINIC ACID (three-letter code: SIN) (formula: $C_4H_6O_4$) (labeled as "Ligand of Interest" by author).

CONFIDENTIAL VALIDATION REPORT

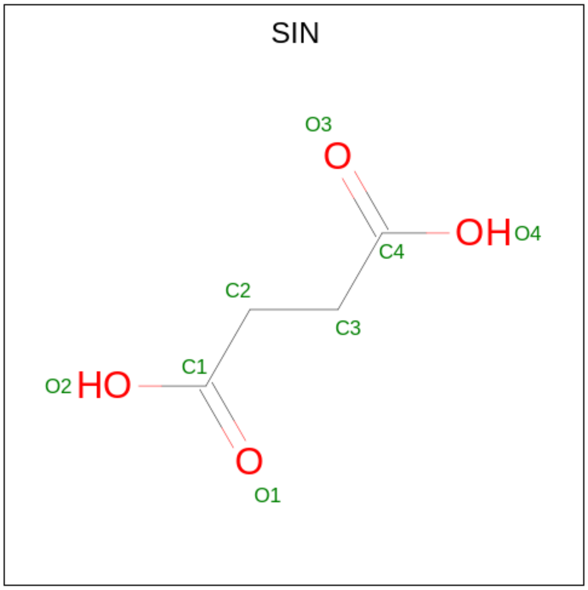

Mol	Chain	Residues	Atoms	ZeroOcc	AltConf
2	A	1	Total C O<br>8 4 4	0	0

- Molecule 3 is MAGNESIUM ION (three-letter code: MG) (formula: Mg) (labeled as "Ligand of Interest" by author).

Mol	Chain	Residues	Atoms	ZeroOcc	AltConf
3	B	1	Total Mg<br>1 1	0	0
3	A	1	Total Mg<br>1 1	0	0

- Molecule 4 is 2-methylidenebutanedioic acid (three-letter code: ITN) (formula: $C_5H_6O_4$) (labeled as "Ligand of Interest" by author).

CONFIDENTIAL VALIDATION REPORT

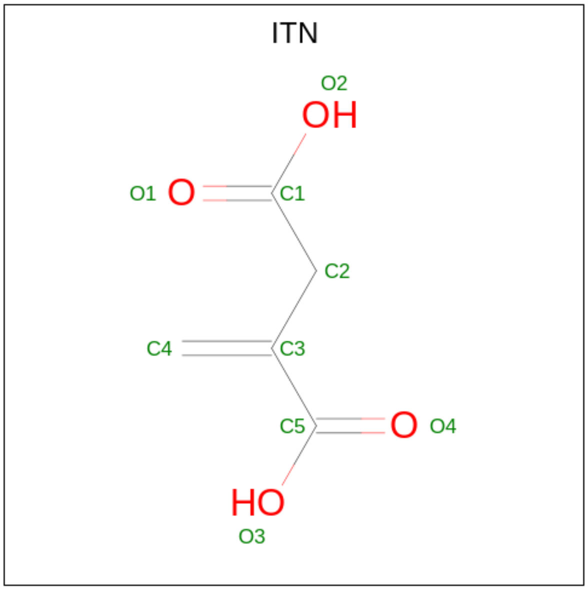

Mol	Chain	Residues	Atoms	ZeroOcc	AltConf
4	B	1	Total C O<br>9 5 4	0	0

- Molecule 5 is water.

Mol	Chain	Residues	Atoms	ZeroOcc	AltConf
5	A	324	Total O<br>324 324	0	0
5	B	317	Total O<br>317 317	0	0

CONFIDENTIAL VALIDATION REPORT

3 Residue-property plots (i)

These plots are drawn for all protein, RNA, DNA and oligosaccharide chains in the entry. The first graphic for a chain summarises the proportions of the various outlier classes displayed in the second graphic. The second graphic shows the sequence view annotated by issues in geometry and electron density. Residues are color-coded according to the number of geometric quality criteria for which they contain at least one outlier: green = 0, yellow = 1, orange = 2 and red = 3 or more. A red dot above a residue indicates a poor fit to the electron density (RSRZ > 2). Stretches of 2 or more consecutive residues without any outlier are shown as a green connector. Residues present in the sample, but not in the model, are shown in grey.

- Molecule 1: Isocitrate lyase

Chain A:

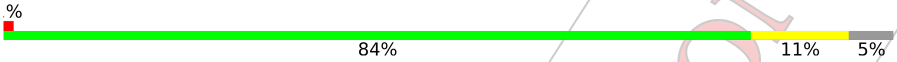

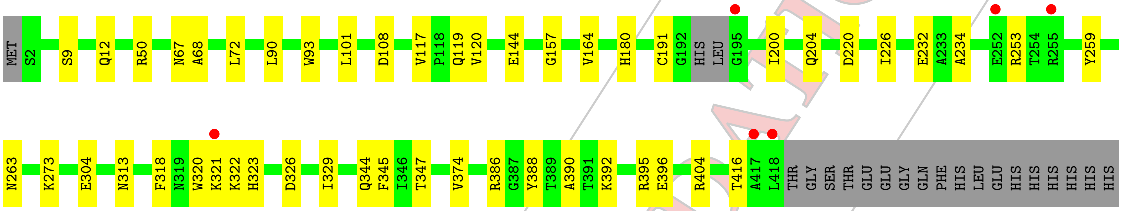

- Molecule 1: Isocitrate lyase

Chain B:

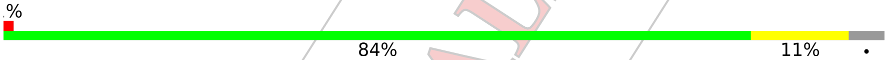

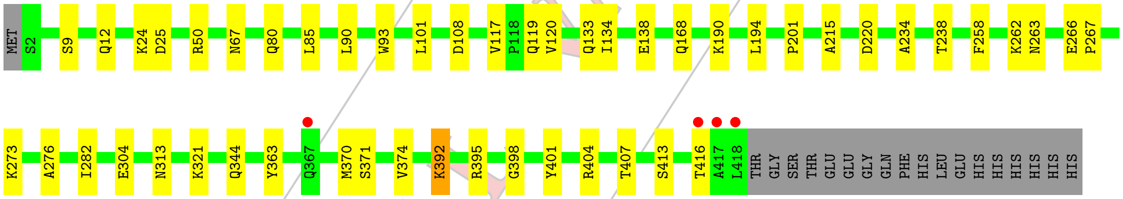

CONFIDENTIAL VALIDATION REPORT

4 Data and refinement statistics (i)

Property	Value	Source
Space group	P 65 2 2	Depositor
Cell constants<br>a, b, c, α, β, γ	131.32Å 131.32Å 284.88Å<br>90.00° 90.00° 120.00°	Depositor
Resolution (Å)	43.03 – 2.58<br>43.03 – 2.58	Depositor<br>EDS
% Data completeness<br>(in resolution range)	92.4 (43.03-2.58)<br>92.4 (43.03-2.58)	Depositor<br>EDS
R_{merge}	0.15	Depositor
R_{sym}	(Not available)	Depositor
$< I/\sigma(I) >$ [1]	3.26 (at 2.58Å)	Xtriage
Refinement program	PHENIX 1.14_3260	Depositor
R, R_{free}	0.154 , 0.194<br>0.154 , 0.194	Depositor<br>DCC
R_{free} test set	2119 reflections (4.92%)	wwPDB-VP
Wilson B-factor (Å^2)	33.0	Xtriage
Anisotropy	0.359	Xtriage
Bulk solvent k_{sol}(e/Å^3), B_{sol}(Å^2)	0.35 , 44.9	EDS
L-test for twinning[2]	$< \|L\| > = 0.45$, $< L^2 > = 0.28$	Xtriage
Estimated twinning fraction	No twinning to report.	Xtriage
F_o,F_c correlation	0.95	EDS
Total number of atoms	7102	wwPDB-VP
Average B, all atoms (Å^2)	32.0	wwPDB-VP

Xtriage's analysis on translational NCS is as follows: *The largest off-origin peak in the Patterson function is 4.92% of the height of the origin peak. No significant pseudotranslation is detected.*

[1] Intensities estimated from amplitudes.
[2] Theoretical values of $< |L| >$, $< L^2 >$ for acentric reflections are 0.5, 0.333 respectively for untwinned datasets, and 0.375, 0.2 for perfectly twinned datasets.

CONFIDENTIAL VALIDATION REPORT

5 Model quality (i)

5.1 Standard geometry (i)

Bond lengths and bond angles in the following residue types are not validated in this section: ITN, MG, SIN

The Z score for a bond length (or angle) is the number of standard deviations the observed value is removed from the expected value. A bond length (or angle) with $|Z| > 5$ is considered an outlier worth inspection. RMSZ is the root-mean-square of all Z scores of the bond lengths (or angles).

Mol	Chain	Bond lengths		Bond angles	
		RMSZ	$\#\|Z\| > 5$	RMSZ	$\#\|Z\| > 5$
1	A	0.45	0/3279	0.61	0/4459
1	B	0.43	0/3301	0.59	0/4491
All	All	0.44	0/6580	0.60	0/8950

There are no bond length outliers.

There are no bond angle outliers.

There are no chirality outliers.

There are no planarity outliers.

5.2 Too-close contacts (i)

In the following table, the Non-H and H(model) columns list the number of non-hydrogen atoms and hydrogen atoms in the chain respectively. The H(added) column lists the number of hydrogen atoms added and optimized by MolProbity. The Clashes column lists the number of clashes within the asymmetric unit, whereas Symm-Clashes lists symmetry related clashes.

Mol	Chain	Non-H	H(model)	H(added)	Clashes	Symm-Clashes
1	A	3211	0	3102	34	0
1	B	3231	0	3120	32	0
2	A	8	0	4	2	0
3	A	1	0	0	0	0
3	B	1	0	0	0	0
4	B	9	0	0	0	0
5	A	324	0	0	1	0
5	B	317	0	0	4	0
All	All	7102	0	6226	64	0

The all-atom clashscore is defined as the number of clashes found per 1000 atoms (including hydrogen atoms). The all-atom clashscore for this structure is 5.

CONFIDENTIAL VALIDATION REPORT

All (64) close contacts within the same asymmetric unit are listed below, sorted by their clash magnitude.

Atom-1	Atom-2	Interatomic distance (Å)	Clash overlap (Å)
1:A:321:LYS:HE3	1:A:326:ASP:OD1	1.63	0.99
1:B:392:LYS:HG3	1:B:395:ARG:HB3	1.65	0.78
1:A:392:LYS:HG3	1:A:395:ARG:HB3	1.65	0.76
1:A:93:TRP:HB2	2:A:501:SIN:H22	1.72	0.72
1:B:190:LYS:HE2	1:B:258:PHE:HE2	1.56	0.69
1:A:395:ARG:NH1	1:A:396:GLU:OE1	2.25	0.69
1:A:200:ILE:HD12	1:A:204:GLN:HB3	1.79	0.63
1:B:50:ARG:NH2	1:B:220:ASP:O	2.32	0.61
1:A:404:ARG:NH2	1:A:416:THR:O	2.30	0.58
1:A:253:ARG:HD3	1:A:259:TYR:CZ	2.40	0.57
1:A:321:LYS:O	1:A:323:HIS:N	2.38	0.57
1:A:67:ASN:HA	1:A:344:GLN:O	2.06	0.55
1:A:234:ALA:O	1:A:263:ASN:HB3	2.06	0.55
1:B:24:LYS:HE2	5:B:842:HOH:O	2.07	0.55
1:A:9:SER:OG	1:A:12:GLN:HG3	2.07	0.55
1:B:392:LYS:HD2	5:B:611:HOH:O	2.05	0.55
1:B:90:LEU:HD11	1:B:120:VAL:HG12	1.89	0.55
1:A:321:LYS:HG2	1:A:329:ILE:CD1	2.37	0.54
1:B:404:ARG:NH2	1:B:416:THR:O	2.36	0.54
1:A:50:ARG:NH2	1:A:220:ASP:O	2.42	0.53
1:A:321:LYS:CE	1:A:326:ASP:OD1	2.48	0.52
1:A:386:ARG:HD2	1:B:363:TYR:CE2	2.46	0.51
1:B:90:LEU:HD22	1:B:117:VAL:HG13	1.92	0.50
1:A:313:ASN:HB3	5:A:793:HOH:O	2.11	0.50
1:B:101:LEU:HD11	1:B:119:GLN:HG3	1.94	0.49
1:B:392:LYS:HE2	1:B:395:ARG:H	1.76	0.49
1:A:273:LYS:HD3	1:A:304:GLU:HG3	1.94	0.49
1:A:318:PHE:CD2	1:A:320:TRP:HA	2.48	0.48
1:B:313:ASN:HB3	5:B:827:HOH:O	2.12	0.48
1:B:234:ALA:O	1:B:263:ASN:HB3	2.13	0.48
1:B:67:ASN:HA	1:B:344:GLN:O	2.14	0.48
1:B:9:SER:OG	1:B:12:GLN:HG3	2.14	0.47
1:B:24:LYS:HE3	1:B:25:ASP:OD2	2.15	0.46
1:B:273:LYS:HD3	1:B:304:GLU:HG3	1.97	0.46
1:B:371:SER:O	1:B:374:VAL:HG22	2.16	0.46
1:A:321:LYS:O	1:A:322:LYS:C	2.54	0.46
1:A:157:GLY:HA3	1:A:164:VAL:HG22	1.97	0.46
1:A:93:TRP:CB	2:A:501:SIN:H22	2.46	0.45
1:A:388:TYR:CZ	1:A:390:ALA:HB3	2.52	0.45
1:A:90:LEU:HD11	1:A:120:VAL:HG12	1.98	0.45

Continued on next page...

CONFIDENTIAL VALIDATION REPORT

Continued from previous page...

Atom-1	Atom-2	Interatomic distance (Å)	Clash overlap (Å)
1:A:200:ILE:HD12	1:A:204:GLN:CB	2.46	0.45
1:A:374:VAL:HG11	1:B:321:LYS:HG3	1.99	0.44
1:B:370:MET:O	1:B:374:VAL:HG13	2.17	0.44
1:B:93:TRP:CD1	1:B:108:ASP:HB2	2.52	0.44
1:A:180:HIS:HB3	1:A:226:ILE:HB	1.99	0.44
1:B:133:GLN:HB3	1:B:401:TYR:CE1	2.51	0.44
1:A:68:ALA:O	1:A:345:PHE:HA	2.18	0.44
1:B:80:GLN:HB3	1:B:85:LEU:HD12	2.00	0.44
1:A:321:LYS:C	1:A:323:HIS:N	2.71	0.43
1:A:93:TRP:CD1	1:A:108:ASP:HB2	2.53	0.43
1:B:134:ILE:HD11	1:B:398:GLY:HA2	2.01	0.43
1:A:232:GLU:O	1:A:263:ASN:HB2	2.19	0.43
1:B:266:GLU:HB3	1:B:267:PRO:HD3	2.01	0.42
1:B:276:ALA:HA	1:B:282:ILE:HD11	2.02	0.42
1:B:138:GLU:OE2	5:B:602:HOH:O	2.21	0.42
1:B:201:PRO:HD3	1:B:238:THR:OG1	2.20	0.41
1:B:168:GLN:HG2	1:B:215:ALA:HB3	2.02	0.41
1:A:101:LEU:HD11	1:A:119:GLN:HG3	2.02	0.41
1:B:194:LEU:HD23	1:B:194:LEU:HA	1.94	0.41
1:B:392:LYS:HD2	1:B:392:LYS:C	2.39	0.41
1:A:313:ASN:OD1	1:A:347:THR:HG23	2.19	0.41
1:B:407:THR:HG23	1:B:413:SER:HB3	2.02	0.41
1:A:321:LYS:C	1:A:323:HIS:H	2.23	0.40
1:A:90:LEU:HD22	1:A:117:VAL:HG13	2.03	0.40

There are no symmetry-related clashes.

5.3 Torsion angles (i)

5.3.1 Protein backbone (i)

In the following table, the Percentiles column shows the percent Ramachandran outliers of the chain as a percentile score with respect to all X-ray entries followed by that with respect to entries of similar resolution.

The Analysed column shows the number of residues for which the backbone conformation was analysed, and the total number of residues.

Mol	Chain	Analysed	Favoured	Allowed	Outliers	Percentiles	
1	A	411/436 (94%)	403 (98%)	8 (2%)	0	100	100

Continued on next page...

CONFIDENTIAL VALIDATION REPORT

Continued from previous page...

Mol	Chain	Analysed	Favoured	Allowed	Outliers	Percentiles	
1	B	415/436 (95%)	407 (98%)	8 (2%)	0	100	100
All	All	826/872 (95%)	810 (98%)	16 (2%)	0	100	100

There are no Ramachandran outliers to report.

5.3.2 Protein sidechains (i)

In the following table, the Percentiles column shows the percent sidechain outliers of the chain as a percentile score with respect to all X-ray entries followed by that with respect to entries of similar resolution.

The Analysed column shows the number of residues for which the sidechain conformation was analysed, and the total number of residues.

Mol	Chain	Analysed	Rotameric	Outliers	Percentiles	
1	A	326/347 (94%)	323 (99%)	3 (1%)	78	90
1	B	329/347 (95%)	327 (99%)	2 (1%)	86	94
All	All	655/694 (94%)	650 (99%)	5 (1%)	81	92

All (5) residues with a non-rotameric sidechain are listed below:

Mol	Chain	Res	Type
1	A	72	LEU
1	A	144	GLU
1	A	191	CYS
1	B	262	LYS
1	B	392	LYS

Some sidechains can be flipped to improve hydrogen bonding and reduce clashes. All (1) such sidechains are listed below:

Mol	Chain	Res	Type
1	B	367	GLN

5.3.3 RNA (i)

There are no RNA molecules in this entry.

CONFIDENTIAL VALIDATION REPORT

5.4 Non-standard residues in protein, DNA, RNA chains (i)

There are no non-standard protein/DNA/RNA residues in this entry.

5.5 Carbohydrates (i)

There are no monosaccharides in this entry.

5.6 Ligand geometry (i)

Of 4 ligands modelled in this entry, 2 are monoatomic - leaving 2 for Mogul analysis.

In the following table, the Counts columns list the number of bonds (or angles) for which Mogul statistics could be retrieved, the number of bonds (or angles) that are observed in the model and the number of bonds (or angles) that are defined in the Chemical Component Dictionary. The Link column lists molecule types, if any, to which the group is linked. The Z score for a bond length (or angle) is the number of standard deviations the observed value is removed from the expected value. A bond length (or angle) with $|Z| > 2$ is considered an outlier worth inspection. RMSZ is the root-mean-square of all Z scores of the bond lengths (or angles).

Mol	Type	Chain	Res	Link	Bond lengths			Bond angles		
					Counts	RMSZ	$\#\|Z\| > 2$	Counts	RMSZ	$\#\|Z\| > 2$
2	SIN	A	501	-	1,7,7	0.01	0	2,8,8	1.98	1 (50%)
4	ITN	B	501	-	2,8,8	4.06	1 (50%)	2,10,10	2.28	2 (100%)

In the following table, the Chirals column lists the number of chiral outliers, the number of chiral centers analysed, the number of these observed in the model and the number defined in the Chemical Component Dictionary. Similar counts are reported in the Torsion and Rings columns. '-' means no outliers of that kind were identified.

Mol	Type	Chain	Res	Link	Chirals	Torsions	Rings
2	SIN	A	501	-	-	1/1/5/5	-
4	ITN	B	501	-	-	2/2/8/8	-

All (1) bond length outliers are listed below:

Mol	Chain	Res	Type	Atoms	Z	Observed(Å)	Ideal(Å)
4	B	501	ITN	C2-C3	5.73	1.55	1.50

All (3) bond angle outliers are listed below:

Mol	Chain	Res	Type	Atoms	Z	Observed(^{o})	Ideal(^{o})
2	A	501	SIN	C3-C2-C1	2.76	117.30	112.67

Continued on next page...

CONFIDENTIAL VALIDATION REPORT

Continued from previous page...

Mol	Chain	Res	Type	Atoms	Z	Observed(^{o})	Ideal(^{o})
4	B	501	ITN	C1-C2-C3	2.41	119.29	116.07
4	B	501	ITN	C2-C3-C4	-2.15	118.53	122.74

There are no chirality outliers.

All (3) torsion outliers are listed below:

Mol	Chain	Res	Type	Atoms
4	B	501	ITN	C1-C2-C3-C5
4	B	501	ITN	C1-C2-C3-C4
2	A	501	SIN	C1-C2-C3-C4

There are no ring outliers.

1 monomer is involved in 2 short contacts:

Mol	Chain	Res	Type	Clashes	Symm-Clashes
2	A	501	SIN	2	0

The following is a two-dimensional graphical depiction of Mogul quality analysis of bond lengths, bond angles, torsion angles, and ring geometry for all instances of the Ligand of Interest. In addition, ligands with molecular weight > 250 and outliers as shown on the validation Tables will also be included. For torsion angles, if less then 5% of the Mogul distribution of torsion angles is within 10 degrees of the torsion angle in question, then that torsion angle is considered an outlier. Any bond that is central to one or more torsion angles identified as an outlier by Mogul will be highlighted in the graph. For rings, the root-mean-square deviation (RMSD) between the ring in question and similar rings identified by Mogul is calculated over all ring torsion angles. If the average RMSD is greater than 60 degrees and the minimal RMSD between the ring in question and any Mogul-identified rings is also greater than 60 degrees, then that ring is considered an outlier. The outliers are highlighted in purple. The color gray indicates Mogul did not find sufficient equivalents in the CSD to analyse the geometry.

CONFIDENTIAL VALIDATION REPORT

Ligand SIN A 501

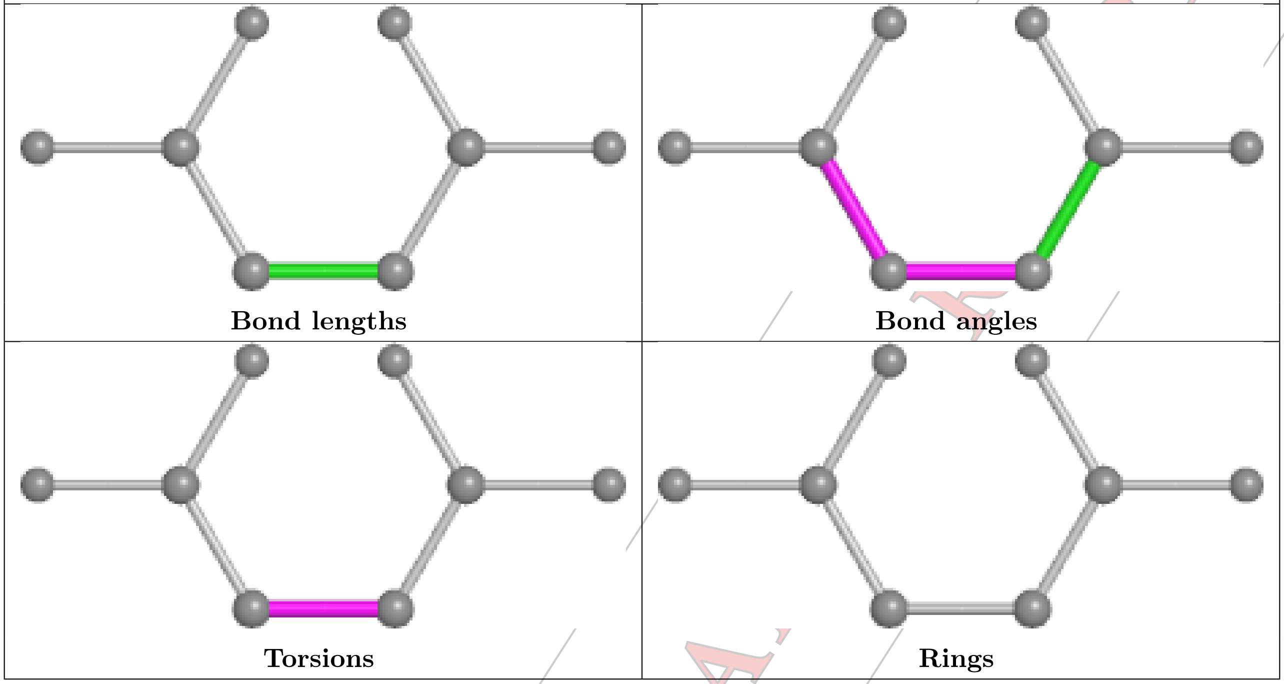

Ligand ITN B 501

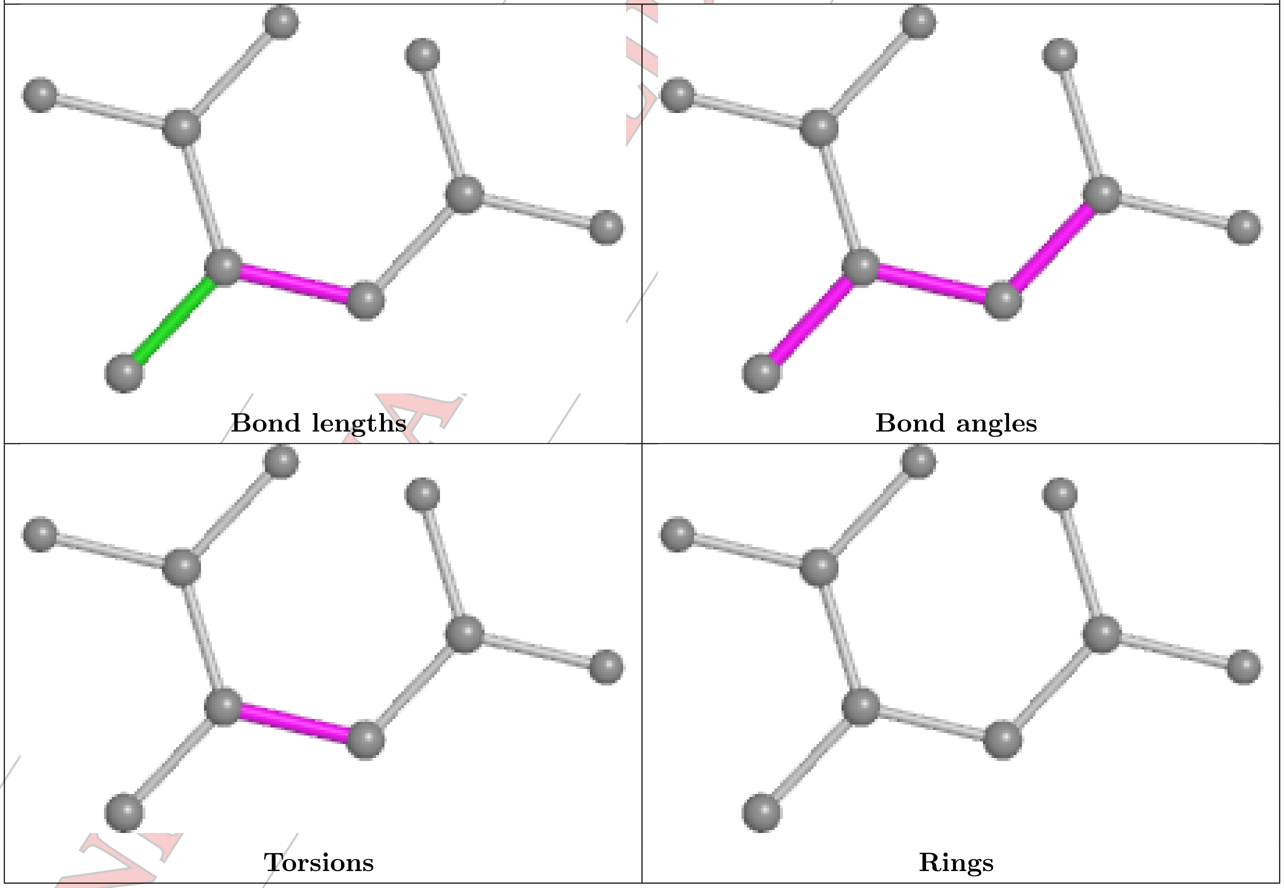

5.7 Other polymers (i)

There are no such residues in this entry.

5.8 Polymer linkage issues (i)

There are no chain breaks in this entry.

CONFIDENTIAL VALIDATION REPORT

6 Fit of model and data (i)

6.1 Protein, DNA and RNA chains (i)

In the following table, the column labelled '#RSRZ> 2' contains the number (and percentage) of RSRZ outliers, followed by percent RSRZ outliers for the chain as percentile scores relative to all X-ray entries and entries of similar resolution. The OWAB column contains the minimum, median, 95th percentile and maximum values of the occupancy-weighted average B-factor per residue. The column labelled 'Q< 0.9' lists the number of (and percentage) of residues with an average occupancy less than 0.9.

Mol	Chain	Analysed	<RSRZ>	#RSRZ>2			OWAB(Å^2)	Q<0.9
1	A	415/436 (95%)	-0.35	6 (1%)	75	73	19, 31, 51, 79	0
1	B	417/436 (95%)	-0.37	4 (0%)	82	81	21, 30, 49, 78	0
All	All	832/872 (95%)	-0.36	10 (1%)	79	77	19, 31, 50, 79	0

All (10) RSRZ outliers are listed below:

Mol	Chain	Res	Type	RSRZ
1	B	417	ALA	5.5
1	B	418	LEU	4.4
1	A	195	GLY	3.2
1	B	416	THR	2.9
1	A	418	LEU	2.8
1	A	417	ALA	2.6
1	A	255	ARG	2.4
1	B	367	GLN	2.1
1	A	252	GLU	2.0
1	A	321	LYS	2.0

6.2 Non-standard residues in protein, DNA, RNA chains (i)

There are no non-standard protein/DNA/RNA residues in this entry.

6.3 Carbohydrates (i)

There are no monosaccharides in this entry.

CONFIDENTIAL VALIDATION REPORT

6.4 Ligands (i)

In the following table, the Atoms column lists the number of modelled atoms in the group and the number defined in the chemical component dictionary. The B-factors column lists the minimum, median, 95th percentile and maximum values of B factors of atoms in the group. The column labelled 'Q< 0.9' lists the number of atoms with occupancy less than 0.9.

Mol	Type	Chain	Res	Atoms	RSCC	RSR	B-factors($Å^2$)	Q<0.9
2	SIN	A	501	8/8	0.87	0.26	34,39,45,53	0
4	ITN	B	501	9/9	0.91	0.18	22,34,48,53	0
3	MG	B	502	1/1	0.99	0.24	22,22,22,22	0
3	MG	A	502	1/1	0.99	0.23	22,22,22,22	0

The following is a graphical depiction of the model fit to experimental electron density of all instances of the Ligand of Interest. In addition, ligands with molecular weight > 250 and outliers as shown on the geometry validation Tables will also be included. Each fit is shown from different orientation to approximate a three-dimensional view.

Electron density around SIN A 501:

$2mF_o$-DF_c (at 0.7 rmsd) in gray
mF_o-DF_c (at 3 rmsd) in purple (negative)
and green (positive)

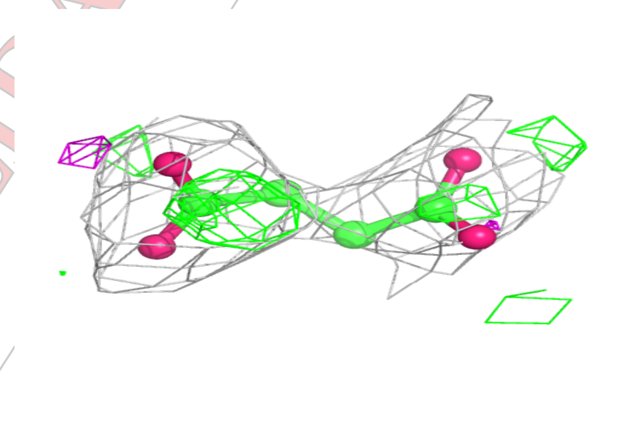

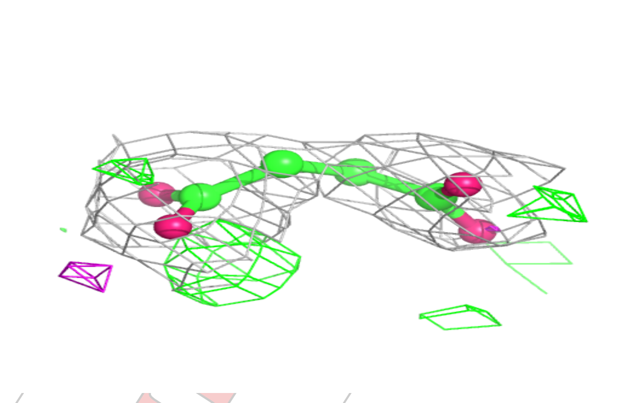

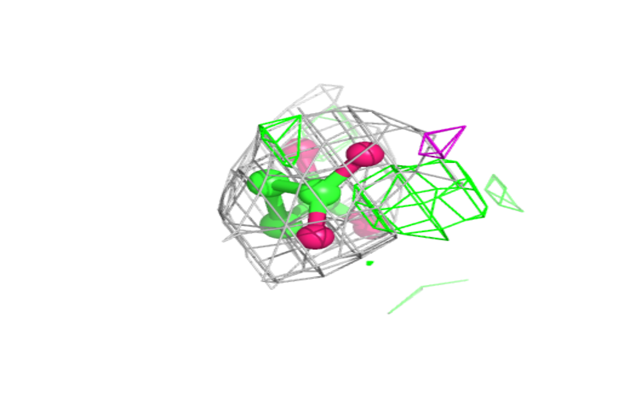

CONFIDENTIAL VALIDATION REPORT

Electron density around ITN B 501:

$2mF_o$-DF_c (at 0.7 rmsd) in gray
mF_o-DF_c (at 3 rmsd) in purple (negative)
and green (positive)

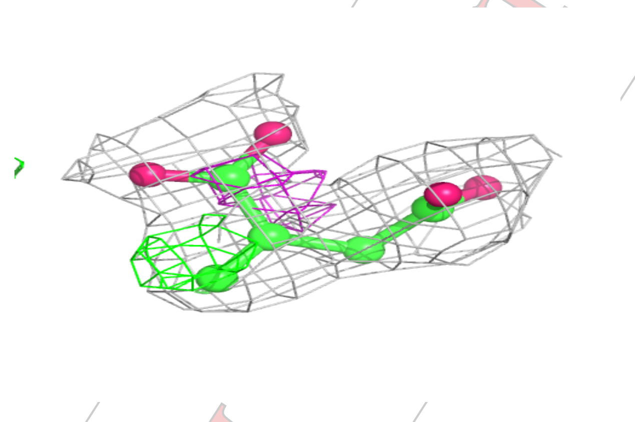

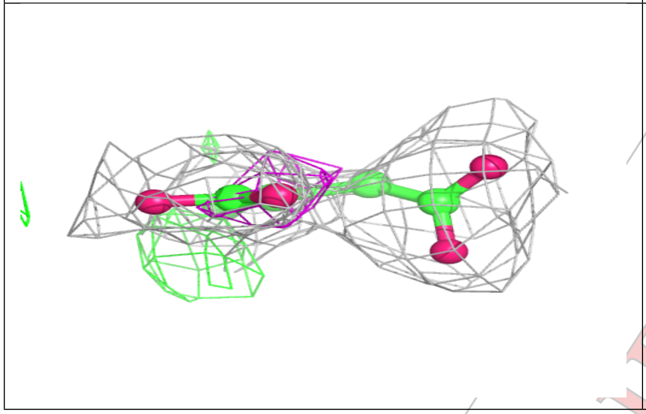

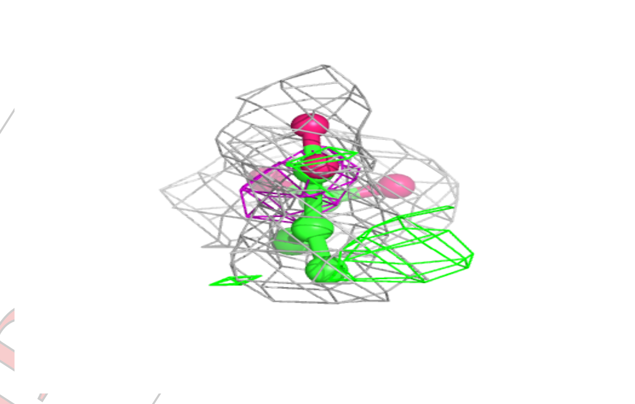

Electron density around MG B 502:

$2mF_o$-DF_c (at 0.7 rmsd) in gray
mF_o-DF_c (at 3 rmsd) in purple (negative)
and green (positive)

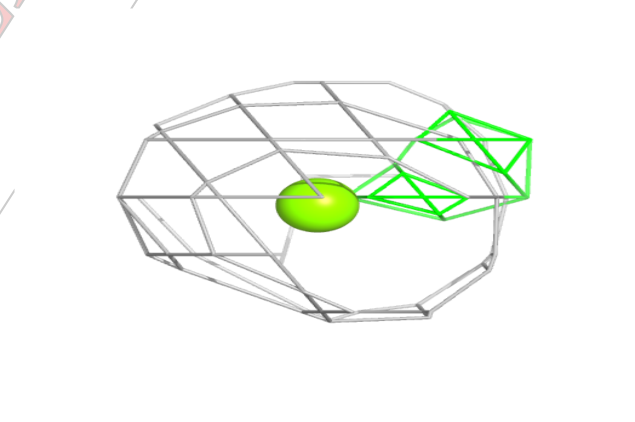

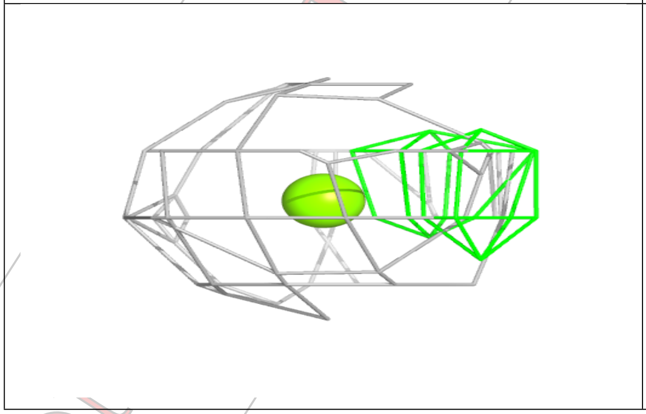

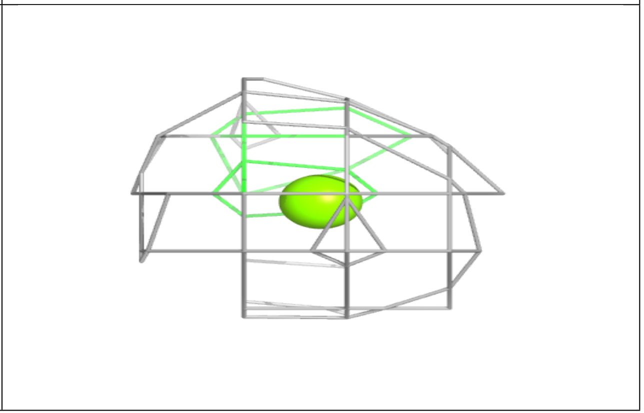

Electron density around MG A 502:

$2mF_o$-DF_c (at 0.7 rmsd) in gray
mF_o-DF_c (at 3 rmsd) in purple (negative)
and green (positive)

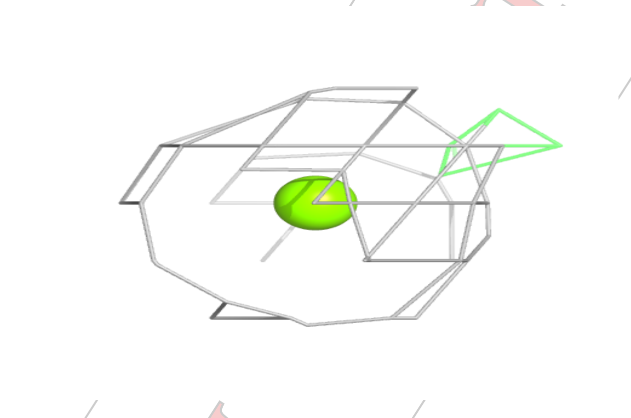

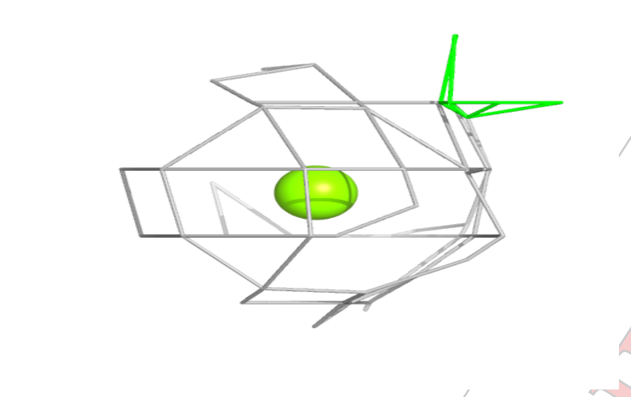

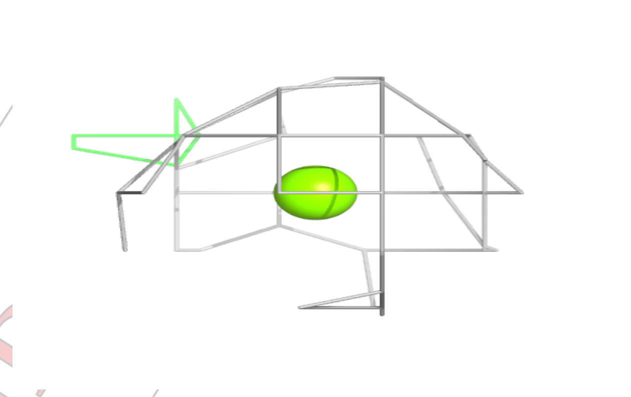

6.5 Other polymers ⓘ

There are no such residues in this entry.

CONFIDENTIAL VALI
